# The CNS microvascular pericyte: pericyte-astrocyte crosstalk in the regulation of tissue survival

**DOI:** 10.1186/2045-8118-8-8

**Published:** 2011-01-18

**Authors:** Drew Bonkowski, Vladimir Katyshev, Roumen D Balabanov, Andre Borisov, Paula Dore-Duffy

**Affiliations:** 1 Department of Neurology, Wayne State University School of Medicine, 421 East Canfield Road, Detroit, Michigan 48201, USA; 2 Department of Neuroscience, University of Michigan, Ann Arbor, Michigan, USA; 3 Department of Neurology Wayne State University School of Medicine, Detroit, Michigan 48201, USA; 4 Department of Neurology, Rush University Medical Center, Chicago, Illinois, 60612, USA; 5 Department of Biology, Wayne State University, Detroit, Michigan 48201, USA

## Abstract

The French scientist Charles Benjamin Rouget identified the pericyte nearly 140 years ago. Since that time the role of the pericyte in vascular function has been difficult to elucidate. It was not until the development of techniques to isolate and culture pericytes that scientists have begun to understand the true impact of this unique cell in the maintenance of tissue homeostasis. In the brain the pericyte is an integral cellular component of the blood-brain barrier and, together with other cells of the neurovascular unit (endothelial cells, astrocytes and neurons) the pericyte makes fine-tuned regulatory adjustments and adaptations to promote tissue survival. These regulatory changes involve trans-cellular communication networks between cells. In this review we consider evidence for cell-to-cell crosstalk between pericytes and astrocytes during development and in adult brain.

## Review

### Introduction

The blood-brain barrier (BBB) regulates the passage of nutrients, essential components, proteins, chemical substances and microscopic organisms between the bloodstream and the parenchymal tissue. The anatomical constituents of the BBB are the endothelial cells (EC), pericytes, and basal lamina (matrix proteins) that with astrocytes, neurons, and possibly other glial cells, comprise the neurovascular unit [1]. Together, the cells of the neurovascular unit adapt to environmental changes and make fine-tuned regulatory decisions that maintain homeostasis and promote tissue survival [1-4]. Nowhere is such tight regulation more important than in the brain where bioenergetic and metabolic homeostasis is integral for neuronal survival [1,2]. Dysregulation at the neurovascular level is linked to many common human CNS pathologies, making the unit a potential target for therapeutic intervention [1-4].

The role of the CNS pericyte in the neurovascular unit is still unclear although with improved culturing techniques and the use of genetically engineered animals it has become increasingly clear that pericytes are integral to BBB function [3,4]. It is known that pericytes are highly complex regulatory cells that communicate with ECs and other cells of the neurovascular unit such as neurons by direct physical contact and through autocrine and paracrine signaling pathways [3-9]. However, while there is abundant indirect evidence there is little direct evidence of pericyte-astrocyte communication. In this review we will summarize what is known about pericyte-astrocyte transcellular communication. We will discuss vascular development, BBB function and regulation of the neurovascular unit. The potential role of pericyte-astrocyte crosstalk in development of disease pathology will also be covered.

#### 1. Pericytes

Pericytes were described over 100 years ago in 1873 by the French scientist Charles-Marie Benjamin Rouget and dubbed the Rouget cell [10]. The Rouget cell was renamed in the early 1900s to reflect its anatomical location with in the microvasculature abluminal to the EC and luminal to parenchymal cells (reviewed in [11]). In the brain, pericytes are located in pre-capillary arterioles, capillaries and post capillary venules. They synthesize and deposit elements of the basal lamina and are totally surrounded by this vascular compartment [3-7]. Pericytes are local regulatory cells that are important for the maintenance of vascular homeostasis and hemostasis, and are a source of adult pluripotent stem cells [12,13]. Pericytes are important cellular constituents of the BBB and actively communicate with other cells of the neurovascular unit such as EC, astrocytes, and neurons. While the communication networks involving pericytes and endothelial cells have been considered in a number of publications, less is known of the cell-to-cell communication involving astrocytes.

Since its original discovery there has been much confusion and controversy about the pericyte as reflected by the numerous conflicting publications and definitions found in the literature. The pericyte has been referred to as: a) A contractile cell that surrounds the capillary in a tunic-like fashion [10]. b) A branching contractile cell on the abluminal wall of a capillary. c) A peculiar elongated, contractile cell wrapped around pre-capillary arterioles 'outside' the basement membrane [14]. d) A relatively undifferentiated connective tissue cell in the capillaries or other small blood vessels also called the adventitial cell [15]. e) A smooth muscle/pericyte or smooth muscle cell of the capillaries [16,17]. f) A broad flat cell with slender projections that wraps around the capillaries [17]. g) A mesenchymal stem-like cell, associated with the walls of small blood vessels. As a relatively undifferentiated cell, it serves to support these vessels, but it can differentiate into a fibroblast, smooth muscle cell, or macrophage as well other cell populations if required [3,5-7,12,13,18,19]. h) A pluripotent or pericyte progenitor cell [13].

In the mature CNS capillary, the pericyte is located between the EC and parenchymal astrocytes and neurons and is surrounded by the basal lamina [3-7]. Pericytes have a prominent round nucleus that clearly differs in shape from the elongated cigar shaped nucleus of the EC. The pericyte extends long processes that extend over the vessel wall. The morphological pattern of projections appears to be somewhat heterogeneous [13]. Pericyte projections can extend around the capillary as originally described by Rouget [10]. The classic wrapping pattern is also somewhat heterogeneous. The most common association of the pericyte with the capillary is one in which the pericyte processes are large and broad and span a continuous surface of the vessel. Alternatively these processes may form finger-like projections that are more confined and ensheath a more finite portion of the vessel surface. A third pattern of pericyte orientation in the microvessel involves a retraction of projections with protrusion of the cells away from the capillaries this represents a migrating pericyte [20]. Pericytes may also extend along the axis of the capillary. It is unclear whether morphological differences in pericyte coverage represent changes in pericyte function. It is clear however that pericytes can migrate away from the capillary surface during angiogenesis, in response to stress stimuli [20-24] and possibly under other conditions [13]. In normal capillaries the wrapping pattern predominates but under pathological conditions the migrating patterns increase [24] and are associated with upregulation of cell surface proteases [20,21,25].

The CNS pericyte is surrounded by the basal lamina on all sides. During development and during angiogenesis the pericyte, assisted by nearby astrocytes discussed below, deposits basal lamina components [3,4,7,13,25-32]. Even extended pericyte projections, observed using electron microscopy, have a thin layer of basal lamina. The basal lamina has been shown to become thicker or thinner in response to stress stimuli [24,33-36]. Changes in the basal lamina can be directly associated with pericyte expression of proteases [20,24,25,37-41] and ultimate migration from its vascular location [20,33,34,42].

The intact basal lamina may provide anchoring and structural integrity to the capillary but it may also be involved in regulation of pericyte function and differentiation. It seems intuitive that there must be a reason why the pericyte is surrounded by laminal proteins. Αvβ8 integrin is important in neurovascular cell adhesion [27,43]. Pericytes encased in the basal lamina or exposed to laminal proteins do not usually differentiate (Dore-Duffy, unpublished observations). Thus migration through the basal lamina is necessary before pericytes can function in their stem cell capacity [13]. Regulation at the level of the basal lamina may also be integral to vascular adaptability to an ever-changing environment and to pericyte signaling mechanisms [24].

In its pericapillary location, the pericyte may signal nearby ECs [3,4,13,40,44,45], astrocytes [46,47], neurons, smooth muscle cells and perhaps other pericytes [13]. Pericyte-EC contacts include peg and socket arrangements [8,48] and gap junctions [49-52]. Gap junctions allow pericytes to communicate with ECs through the exchange of ions and small molecules. Peg-and-socket contacts enable pericytes to penetrate through the basal lamina and make contact with other cells and nearby vessels [8,48,53]. Junction complexes including adhesion plaques also support transmission of contractile forces from pericytes to other cells. Pericyte gap junctions contain N-cadherin, a variety of adhesion molecules, β-catenin, extracellular matrix (ECM) molecules such as fibronectin, and a number of integrins [50,51]. Thus, pericytes are involved in highly complex signaling cascades that enable this cell to respond to changes in the microenvironment. However, it is unclear whether gap junctions and peg and socket contacts are naturally present or whether they are initiated during changes in functional activity. For example, it is known that pericytes interdigitate with ECs during the early phases of angiogenesis and with neurons during the maturation of newly-formed vessels [54]. These sites of communication are altered under pathological conditions. During cerebral edema or diabetes, gap junctions are substantially decreased or disrupted in retinal pericytes [54-57]. Diabetes-induced changes in gap junctions may be regulated by high glucose [56-58]. Pericyte-EC communication via gap junctions is fundamental to the adaptive responses to compromised bioenergetic homeostasis [58]. Crosstalk between ECs, pericytes, as well as astrocytes is involved in regulation of insulin transport [47]. Pericyte/EC crosstalk is also integral to physiological angiogenesis [59], and is likely to be important in adaptation to hypoxic injury and focal capillary contractility.

#### 2. Pericytes and astrocytes during vasculogenesis and angiogenesis

Vasculogenesis is the formation of new blood-vessels by differentiation of vascular precursor cells during development. During retinal development, a role for astrocyte-pericyte communication has been established. Retinal vascularization begins in the inner retinal layer and sprouts radially from the optic nerve to reach the periphery of the retina [60]. Subsequently, retinal vessels sprout into the deep retinal layer to vascularize three parallel nerve fiber layers and two plexiform layers. Experiments using retinal models have tested the hypothesis that astrocytes and pericytes influence this process by affecting the composition of the extracellular matrix. Fibronectin, but not laminin, is expressed in zones of vasculogenesis immediately prior to vessel formation. At this time astrocytes and pericytes spread into the tissue and may be involved in the initiation of vasculogenesis. In fact, it is unclear whether platelet-derived growth factor receptors PDGFβR+/PDGFαR+ pericytes and/or PDGFαR+ immature astrocytes are the regulating cell type [60]. Increased amounts of fibronectin mRNA suggest that fibronectin is synthesized by cells within this same region. Pericytes are known to synthesize most extracellular matrix protein components [61]. Differentiation of endothelial cells is correlated with the appearance of pericytes in the vessel wall and laminin in the vascular basement membrane. Astrocyte-conditioned medium stimulates fibronectin expression by both primary endothelial cells and pericytes [62]. Astrocyte-induced pericyte synthesis of fibronectin and perhaps laminin is an essential step in the initiation of retinal vasculogenesis [63]. It is, however, unclear whether a similar mechanism functions in the brain.

Vascularization in the brain during development is derived from a preformed perineural vascular plexus and occurs almost exclusively through sprouting angiogenesis that starts at embryonic day 9 in vertebrates [63]. Angiogenesis is the formation of new vessels from existing vessels. Sprouting angiogenesis is an invasive process that involves proteolytic activities required for degradation of the basal lamina, with pericyte and endothelial migration through tissue matrix [63]. During vessel formation, the recruitment of pericytes and astrocytes to newly-forming vascular tubes is closely associated with the formation of tight junctions. At P4, invading vessels are in direct contact with pericytes, but not with astrocytes. With the progression of development, foot processes of astrocytes are gathered around retinal vessels and the maturation of tight junction ZO-1 in endothelial cells is more clearly defined [64]. Tight junctions can be formed with contact from pericytes without the ensheathment of the astrocytic foot processes. From *in vitro *studies it is clear that pericytes induce synthesis of both occludin and claudin through the release of angiopoetin-1 [65]. Thus tight junction formation in the BBB could be developed, in part, by cellular interactions between EC and pericytes followed by astrocytes (as reviewed in [66]). The expression of ZO-1 is augmented by astrocytes [67]. Moreover, ZO-1 as well as occludin or claudin together may be biological indicators of barrier maturation [67]. The role of the pericyte in BBB function will be discussed below.

Studies using glial fibrillary acidic protein (GFAP)-knockout mice (GFAP-/-) indicate that, although astrocyte foot interactions with the microvasculature are missing, neovascularization and the formation of tight junctions as well as other CNS morphology appears to be natural [68-70]. Astrocyte neuronal interactions are disrupted [70]. These data suggest that astrocyte interaction with the capillaries or neurons is not essential for tight junction formation. Of interest is that GFAP-/- mice are prone to hemorrhagic injury [71]. Similar observations were observed by McCarthy and colleagues [44] in studies using animals lacking alphav integrins. In contrast, administration of the gliotoxin 6-aminonicotinamide (6-AN) to chick embryos during both early and late embryonic development showed a good correlation between perivascular glial depletion and BBB impairment suggesting that astroglia do in fact play a role in BBB prenatal differentiation and must in some way contribute to vascular function [72]. Taken together the results suggest that, while astrocyte formation of end-feet is not essential for tight junction formation, astrocytes are an important component contributing to normal vessel structural integrity. These results may also indicate that pericytes or other CNS cellular components can compensate for the lack of direct astrocyte signaling.

**Figure 1:**
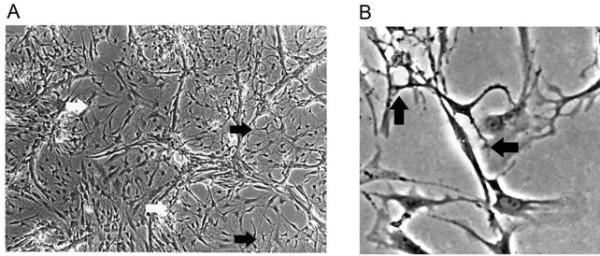
**Induction of angiogenesis in triple co-cultures of ECs, pericytes and astrocytes**. Primary cells were co-cultured at a ratio of one pericyte to five ECs and to five astrocytes. Cultures were exposed to hypoxia (10%) to induce angiogenesis (A). Tube formation in triple cultures involved crosstalk between astrocytes and pericytes. White arrows point to pericytes and black arrows point to astrocytes. Photograph was taken using phase contrast at 10×. In (B) we show a close up of an astrocyte making contact with both elongating EC and pericytes. The black arrows point to astrocyte contacts with an elongating EC (left) and one of the two contacts made with a pericyte (right). Pericytes have fewer projections and are more spread out than the astrocytes.

Using *in vitro *model systems to study vasculogenesis, scientists have learned that both pericytes and astrocytes are involved in formation of capillary structures. Both cell types form endothelial connections with newly forming vessels (Figure 1) realigning themselves according to the same arrangement seen *in vivo*. Pericytes in co-culture associate with EC more rapidly than astrocytes. In multi-cellular systems involving pericytes, astrocytes, and EC newly forming tubes have the three dimensional structure of a vessel and exhibit both a lumen and tight junctions. Tube formation in triple cultures is more rapid than that observed in EC/pericyte or EC/astrocyte, co-cultures. The triple co-culture of pericytes and astrocytes with ECs is thought by most investigators to represent a better model system to study the BBB [73-76]. This may be in part due to the role of agrin, aquaporin 4, and astrocyte polarity in the BBB [77].

The process of vasculogenesis and differentiation is a complex system that involves crosstalk between numerous cells and is regulated by a number of signaling pathways.

Cell surface proteases expressed on both EC and pericytes exert additional subtle functions in sprouting angiogenesis. These functions involve membrane type-1 matrix metalloproteinase (MT1-MMP), other MMPs, and ADAMs (a disintegrin and metalloproteinase domain) [78]. Proteases modulate the balance between pro- and anti-angiogenic factors by activation of growth factors and chemokines, shedding of chemokines and cytokines from membrane-bound precursors [78], and generation of (matrix) protein fragments that inhibit or activate angiogenesis. Furthermore, they participate in the recruitment of leukocytes and progenitor cells, which contribute to the initiation and progression of angiogenesis. Pericytes are involved in the initiation as well as termination of angiogenesis (as reviewed in [59]). At the initiation of angiogenesis, pericytes are involved in induction of endothelial activation accompanied by augmentation of a variety of proteases [40], adhesion molecules and proteoglycans [79]. Astrocyte expression of tumor necrosis factor converting enzyme (TACE/ADAM-17) may facilitate pericyte PDGFβR signaling mechanisms [80]. Alternatively, at maturation the recruitment of pericytes to the newly-formed endothelial tubes is accompanied by silencing of MMP activities [81]. Recruitment to the newly-formed vessel with termination of angiogenesis involves PDGFβ [82] as well as transforming growth factor beta (TGFβ) [83]. PDGFβ is thought to be essential for the retention of pericytes in the newly-formed vessels [84,85]. Ablation of PDGFβ results in an embryonic lethal mutation associated with multiple vascular abnormalities [82,86,87].

### 3. Pericytes, astrocytes and BBB function

#### 3.1 Contractility

The concept that pericytes regulate blood flow at the capillary level was originally proposed by Steinach and Kahn in 1903 [88] and Ni in 1922 [89]. Both scientific groups studied the effects of electrical stimulation or exposure to toxic stimuli on capillary diameter. Doré reviewed this area in 1923 [11]. As stated by Doré, [11]: "Until a few years ago the capillaries were regarded as elastic tubes undergoing passive distension in accordance with the general blood pressure, the state of contraction or dilatation of the supplying arterioles, and the nutrition of the vascular walls". The concepts put forward in this 1923 review are on target with exciting more-recently published data [90-92].

CNS pericytes have receptors for a large number of vasoactive signaling molecules [3,93-95] suggesting that they have the capacity to be involved in cerebrovascular autoregulation. Expression of alpha muscle actin (**α**SMA) and the intermediate filament desmin, two proteins found in smooth muscle cells, as well as their adherence to the endovascular cells make them potential candidates for the regulation of capillary diameter and focal capillary blood flow [3,8,89,91,96-99]. Electrical stimulation of retinal and cerebellar pericytes is reported to evoke a localized capillary constriction [91,100]. ATP in the retina or noradrenaline in the cerebellum also results in constriction of capillaries by pericytes. Glutamate reverses the constriction produced by noradrenaline [100].

Following simulated ischemia and traumatic brain injury (TBI), capillary pericytes are induced to express αSMA and upregulation of pericyte αSMA is correlated with a focal decrease in capillary diameter [100]. Upregulation of muscle actin was mediated by endothelin-1. Other investigators have shown that capillary contraction can be directly linked to metabolic need [101,102]. Exposure to substances that increase pericyte calcium level induces vessel contraction with a concomitant decrease in the capillary lumen diameter [101-103]. The contractile response appears to involve a cascade of events resulting in the inhibition of Na+/Ca2+ exchangers on the EC [103]. Hypoxia, which closes gap junctions, switches the effect of lactate from contraction to relaxation. Thus, pericyte function may be linked with local vascular adaptation to changes in bioenergetic requirements and is intimately linked to astrocyte function.

The calcium-dependent transcription factor NFATc3 is a member of the nuclear factor of activated T cells (NFAT)-family of transcription factors. NFATc3 is critical for embryonic vascular development and differentiation. Filosa and colleagues investigated the role of glutamate in control of NFATc3 regulation in pericytes [104]. Coronal cortical slices from neonatal rats were subjected to electrical field stimulation or were treated with glutamate receptor agonist (+/-)-1-aminocyclopentane-trans-1,3-dicarboxylic acid (t-ACPD). Electrical field stimulation induced NFATc3 nuclear accumulation in pericytes and astrocytes. The response in pericytes was dependent on metabotropic glutamate receptor (mGluR) activation. NFATc3 nuclear accumulation in pericytes was prevented when astrocytic function was abolished with the gliotoxin L-alpha-aminoadipate. Results suggest that astrocyte glutamate, *via *mGluR activation, may regulate gene transcription in pluripotent vascular pericytes [104].

#### 3.2 BBB Permeability

It has been known for decades that the CNS tissue microenvironment provides the cues for BBB induction and differentiation (as reviewed in [3,7,90,105]). Development of the BBB (barrier genesis) is the result of coordinated molecular signaling at the neurovascular interface [106]. Only recently has the canonical Wnt/beta-catenin pathway and the Wnt7a/7b growth factors been implicated in CNS angiogenesis and in BBB induction [86,105-108]. This pathway interacts with other pathways that are crucial for vascular development such as VEGF. Wnt/beta-catenin pathways enhance pericyte mesenchymal differentiation in the presence of TGF-beta3, as demonstrated by increased Sox-9 expression and glycosaminoglycan release into the extracellular matrix. In contrast, transduction of pericytes with a recombinant adenovirus encoding dominant-negative T-cell factor-4 blocked Wnt/beta-catenin signaling and inhibited pericyte differentiation to chondrocytes, leading to reduced Sox-9, reduced type II collagen expression and reduced glycosaminoglycan accumulation [105,107]. These data demonstrate that TGF-beta3 induces the chondrogenic differentiation of pericytes by inducing Wnt/beta-catenin signaling and T-cell factor-induced gene transcription. In response to injury, the Wnt/beta catenin signaling pathway is enhanced in proliferating NG2 chondroitin sulphate proteoglycan positive cells undergoing differentiation to NG2 glia [109]. It is unclear whether induction of Wnt/beta-catenin signaling in pericytes is responsible for the regulation of the BBB by augmenting the number of NG2+ astrocyte subsets.

Pericytes play a key role in vascular remodeling during development and in the adult. Considerable insight into pericyte biology during development has arisen from studies employing genetically-manipulated mice with disrupted PDGFβ/platelet PDGFβR signaling [84-87,110]. During development, EC-secreted PDGFβ binds to pericyte PDGFβR receptors located on the pericyte plasma membrane resulting in dimerization of the receptor [111]. Pericytes themselves produce PDGFβ and thus may signal in an autocrine fashion [13]. PDGF stimulates the proliferation, migration, and recruitment of pericytes to the vascular wall of newly-formed blood vessels [10,14,15,83-87,112]. PDGFβ and PDGFβR knockout mice have diminished pericyte numbers and a lack of pericyte coverage [87]. The mutation is embryonic lethal with abnormal vasculogenesis [87,112]. Loss of pericytes also results in loss of BBB integrity during embryogenesis [113] clearly showing that pericytes are essential to the differentiation of the BBB during development and have an important role in regulating the BBB [114]. The exact mechanisms are still somewhat unclear as pericytes have been shown to both induce permeability and to inhibit permeability during development [113-115].

PDGFβ and PDGFβR interactions are also involved in pericyte-mediated regulation of vascular function in the adult brain and have a pivotal role in the regulation of the cerebral microcirculation. However, the functional roles of CNS pericytes in the adult and aging brain are less well understood. In the adult animal, it is generally accepted that pericyte association with the microvessel is essential to vascular integrity and that loss of pericytes leads to an increase in vascular leakage and altered vascular function. However, loss of pericytes does not always result in a permanent loss of function and does not always result in disease. Pericytes migrate naturally during the early phases of physiological angiogenesis to make way for growing sprouts [116-119], or in response to stress or injury [20]. Migration from the microvessels following TBI for example, is thought to promote survival as pericytes remaining in their vascular location show signs of degenerative activity [20]. Migration from the vessel involves upregulation of cell surface proteases [20]. Newly-forming vessels both in adult angiogenesis and neovascularization are characterized by increased leakiness. This is transient under normal circumstances but is abnormal in conditions such as pathological angiogenesis. During angiogenesis there is co-migration of pericytes with EC sprouts [116-120] and concomitant proliferation of migrated cells before renewed pericyte coverage and termination of angiogenesis [59]. Pericytes may also be recruited from the bone marrow in adult mice and may have a role in initiation as well as termination of angiogenesis [84,120]. Renewed pericyte coverage restores vascular integrity. These observations are supported by data derived using *in vitro *models of angiogenesis [121-123]. Lack of pericyte termination of angiogenesis alters vascular function and may promote vascular leakiness in part by structural problems with tight junctions [113,114]. Pericytes may also be essential to regulation of transport mechanisms [86,108]. Taken together it is easy to speculate that loss of pericyte function may result in the induction of edema and inflammation [3-7,113,114].

**Figure 2:**
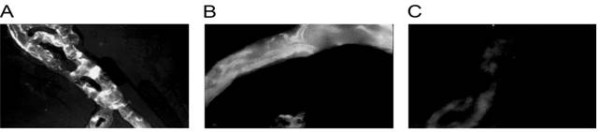
**Capillaries [2-6 micron diameter determined by Image J software] were isolated from wild type C57BL/6 mice (A), GFAP -/+ mice (B), and GFAP -/- mice (C) (Jackson Laboratory)**. Freshly isolated capillaries were allowed to adhere to coverslips fixed and stained for the expression of GFAP (Santa Cruz Biotechnology Inc). Capillaries were visualized on a Leitz fluorescent microscope at 40× and 100×.

**Figure 3:**
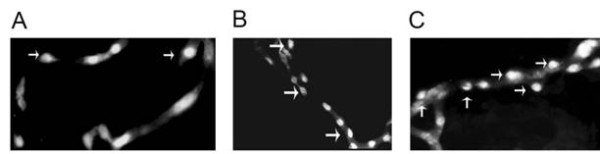
**Pericyte-endothelial cell ratios were determined by isolation of capillaries [2-4 micron diameter determined by Image J software] from wild type B6 mice (A); heterozygous GFAP -/+ mice (B) and knockout GFAP -/- mice (C)**. Freshly isolated capillaries were stained with the fluorescent nuclear dye 4',6-diamidino-2-phenylindole [DAPI]. Round nuclei (pericytes) and elongated nuclei (EC) were counted. Numbers are shown in Table 1. Arrows point to round pericyte nuclei.

Is there a role for astrocytes in maintenance of BBB function? Certainly the astrocytes provide a structural barrier that helps promote vascular integrity. As discussed, microvessels from the GFAP knockout mouse that have no astrocyte end-feet have a tendency to form micro-hemorrhages despite the presence of normal tight junctions [68,69]. The interaction of astrocytes with the capillary appears to have an ordered structure as seen in Figure 2a in wild type capillaries. GFAP staining seems to have a fish net orientation that may assist in promoting vascular integrity. Heterozygous animals display a very disordered GFAP staining (Figure 2b). Capillaries from GFAP-/- mice display no staining pattern (Figure 2c). We have found that, to compensate for the loss of astrocyte end-feet, pericytes proliferate. Pericyte to EC ratios are much higher than those observed for wild type mice and for GFAP+/- heterozygotes (Figure 3; Table 1). This is in contrast to data by others [84] using PDGF deficient animals. The presence of astrogliosis may also alter pericyte function [87].

**Table 1:** Pericyte and endothelial cell numbers in freshly-isolated capillaries from wild type and GFAP knockout mice.

mouse	#Pericytes*	# EC*	Fragments counted/exp
Wild type	3 ± 2	13 ± 2	40
GFAP -/-	9 ± 3	3 ± 1	55
GFAP +/-	7 ± 2	10 ± 3	42

* Mean +/- SD, N = 2 experiments

The integral association of PDGF/PDGFR signaling in maintenance of pericyte function, makes it easy to speculate that astrocytes may be able to regulate pericyte function by the modulation of PDGFβ signaling. Controlled synthesis of astrocyte H_2_O_2 _alters PDGF signaling and is neuroprotective in conditions of oxidative stress [124-128]. The expression of truncated PDGFβR inhibits PDGF signaling [129]. Inhibition is also seen with upregulation of PDGFαR [130]. Heparan sulphate controls the diffusion, and thus the availability of PDGFβ [131]. Perlecan, a heparin sulfate proteoglycan, has been reported to modulate the BBB [132] and is produced by astrocytes during the stress response [133]. Astrocyte modulation of pericyte function is seen in the injured retina [60,64].

### 4. Pericyte-astrocyte communication and disease

Under normal conditions, the pericyte is relatively quiescent and is essential for vascular stability. Under conditions of stress or injury, the pericyte undergoes phenotypic and functional changes that may include migration, proliferation or differentiation. How these events that include pericyte reprogramming are coordinated at the molecular level needs to be determined. However, it is clear that pericyte dysfunction or the loss of pericytes is likely to play an important role in the pathogenesis of disease.

Pericyte loss or a reduced pericyte-to-EC ratio may be achieved through: 1) migration of pericytes from their microvascular location under pathological or physiological conditions, 2) pericyte death, 3) reduced pericyte turnover or maintenance, and 4) selective alteration of pericyte recruitment to EC that may be associated with dysregulation of angiogenesis and abnormal PDGFβ signaling. Loss of pericytes with reduced pericyte-to-EC ratios results in a focal increase in permeability. This can be normal as seen in the transient decrease in pericyte-to-EC ratios due to migration of pericytes during endogenous adaptation to chronic mild hypoxia [59,134] or may be abnormal as observed in a number of pathophysiological diseases. Migration may also play a pathogenic role such as that observed in diabetic retinopathy [135]. Decreased pericyte-to-EC ratios have been observed following TBI (20) and stroke [136], multiple sclerosis [137-140], brain tumors [141-143], diabetic retinopathy [135,144], aging [145,146], and in a variety of angiopathies [147]. Pericyte loss may also play a role in Alzheimer's disease, however; enhanced pericyte coverage of some vessels suggests that increased proliferation of pericytes is an adaptation to focal loss of bioenergetic homeostasis [14,140,148,149]. Pericyte loss due to cellular degeneration/apoptosis has been shown in hypertrophic scars, keloids [150,151], early diabetic retinopathy [150,152], brain tumors [153-156], liver cancer [157] hyperglycemia [158], and during development [159]. Premature infants have decreased pericyte coverage [160]. A decreased pericyte-to-EC ratio also observed during vascular regression [160].

Increased pericyte coverage may also be an indicator of vascular dysfunction. Pericyte proliferation has been associated with development of muscularization during pulmonary hypertension and is thought to be due to platelet activating factor [161]. Cochlear pericytes in the *stria vascularis *are markedly affected by acoustic trauma [162]. Levels of the pericyte structural protein, desmin, substantially increase after noise exposure with a corresponding increase in pericyte coverage of vessels. Increased expression levels of desmin were associated with the induction of hypoxia-inducible factor (HIF)-1alpha and the upregulation of vascular endothelial growth factor (VEGF). Inhibition of HIF-1alpha activity decreased VEGF expression levels. Blockade of VEGF activity with SU1498, a VEGF receptor inhibitor, significantly attenuated the expression of desmin in pericytes and may have altered the adaptive process to acoustic stress. Increased pericyte-to-EC ratios are also observed in response to over expression of PDGFβ [163]. The percentage of vessels covered by pericytes is nearly doubled in myelofibrotic bone marrow [164].

Direct evidence for astrocyte augmentation of pericyte coverage is lacking. However, indirect observations suggest that astrocytes may help augment pericyte proliferation through augmentation of PDGFβ levels [165] or release of inflammatory mediators [166] that enhance gliosis. In patients with glaucoma, enhanced proliferation is modest but restricted to NG2+ pericytes [167]. Astrocytes and pericytes both express estrogen receptors [167-169]. Astrocytes have been shown to be a source of estrogen following neuronal injury due to an upregulation of aromatase [167]. Estrogens attenuate PDGFβ signaling in vascular smooth muscle cells [170,171].

On a more subtle level pericyte stem cell activity may be a target for astrocyte modulation. Pericytes have been shown to be multipotent adult stem cells in a number of tissues [12,13]. Primary CNS pericytes are capable of differentiating along both the mesenchymal and neural lineages depending on the culture conditions [[12], as reviewed [13]]. In the absence of serum in the presence of basic fibroblast growth factor (bFGF) cultured adult pericytes form immature NG2+ astrocytes/radial glial cells, NG2+ oligodendrocyte progenitor-like cells and neurons [12]. Thus in response to injury pericytes may undergo neurogenesis. Astrocyte-generated signaling molecules have been shown to augment neurogenesis and differentiation of oligodendrocyte progenitor cells (OPC) [172]. Radial glial cells also retain neurogenic potential [173,174]. During injury responses in the kindled animal, astrocyte activation and induction of neurogenesis is due in part to augmentation of nestin expression in pericytes [175,176]. Following TBI, Wnt/beta-catenin signaling occurs in quiescent NG2 progenitors in the cortex, in subcallosal zone (SCZ) progenitors, and in subependymal cells surrounding the central canal. Initially after TBI, beta-catenin signaling was predominantly increased in a subset of NG2+ progenitors in the cortex [110]. One week following injury, the majority of beta-catenin signaling appeared in reactive astrocytes but not other glial cells. Interestingly, cells with beta-catenin signaling were not generated following spinal cord injury. This suggests that although augmentation of pericyte generated stem cell activity and differentiation may be essential to tissue plasticity in adult animals responses may be tissue specific. Further, differentiations within microvascular locations, as well as other abnormal pericyte responses to injury, are likely to be highly deleterious.

### 5. Pericytes here and now

After its identification by Rouget in the late 1800s, relatively little was published about the pericyte until 1902 when the presence of this intriguing cell was confirmed [11]. The development of tissue culture techniques, as well as genetically-manipulated transgenic models in which pericyte coverage is absent or diminished has stimulated considerable interest and provided invaluable tools needed to study pericyte biology. However, while genetically-modified animals can be an important tool some caution should be used when attributing functional roles from observations derived in knockout animals. Adaptive responses to a genetic modification may also confound the interpretation. One must also consider that the phenotype is a function both of the planned genetic manipulation and of potential adaptive responses to the change. There are a number of examples in the literature that uniquely illustrated this point [177]. Pericytes are integral to the regulation of vascular function as shown in PDGFβ knockouts. However, PDGFα knockout is also embryonic lethal and displays a similar phenotype [178,179], and together with PDGFβ is also integral to postnatal development [180]. In the future more targeted manipulations as well as fate mapping will likely clarify the functional role of pericytes in many tissues.

## Conclusions

In this review we discussed evidence that pericytes and astrocytes undergo direct cell-to-cell communication. We also discuss indirect evidence that suggests that astrocytes and pericytes coordinate BBB function. It is clear that this communication is needed for the maintenance of vascular function and must be integral to endogenous adaptation to injury in the adult neurovascular unit. Loss of pericyte function and/or loss of proper astrocyte contact with the BBB can result in pericyte dysfunction and development of disease. A better understanding of the mechanisms by which pericytes communicate with other cells and how altered communication may result in disease pathology is likely to yield exciting new insights as well as the development of a new therapeutic target in CNS disorders.

## Competing interests

The authors declare that they have no competing interests.

## Authors' contributions

PDD had primary responsibility for the design of experiments discussed within the review and for the organization and conception of the article. RB performed experiments detailed in the figures and tables as well as development of concepts discussed within. VK conducted experiments to provide some of the data within the manuscript as well as helped with its preparation. DB collected and secured data and interpreted material utilized for the review. AB discussed the organization of the review and possible concepts to be included. All authors have reviewed and approved the final version of the manuscript.
